# Hydrogel Preparation Methods and Biomaterials for Wound Dressing

**DOI:** 10.3390/life11101016

**Published:** 2021-09-27

**Authors:** Jingjing Su, Jiankang Li, Jiaheng Liang, Kun Zhang, Jingan Li

**Affiliations:** 1 School of Life Science, Zhengzhou University, 100 Science Road, Zhengzhou 450001, China; jingjingsuzzu@163.com (J.S.); lijkzzu@163.com (J.L.); liangjiaheng1997@163.com (J.L.); 2 School of Materials Science and Engineering, Zhengzhou University, 100 Science Road, Zhengzhou 450001, China

**Keywords:** wound healing, hydrogels, natural biomaterials, synthetic materials

## Abstract

Wounds have become one of the causes of death worldwide. The metabolic disorder of the wound microenvironment can lead to a series of serious symptoms, especially chronic wounds that bring great pain to patients, and there is currently no effective and widely used wound dressing. Therefore, it is important to develop new multifunctional wound dressings. Hydrogel is an ideal dressing candidate because of its 3D structure, good permeability, excellent biocompatibility, and ability to provide a moist environment for wound repair, which overcomes the shortcomings of traditional dressings. This article first briefly introduces the skin wound healing process, then the preparation methods of hydrogel dressings and the characteristics of hydrogel wound dressings made of natural biomaterials and synthetic materials are introduced. Finally, the development prospects and challenges of hydrogel wound dressings are discussed.

## 1. Introduction

The skin is the main defense system of the human body, which can protect the human body from microbial infection and external environmental damage [1,2]. However, due to various internal and external factors, such as physical, chemical, thermal, mechanical, pressure, infection, disease, etc., people often encounter various injuries that cause skin defects. Various wounds of different sizes, depths, and shapes have been formed. They can be divided into acute wounds and chronic wounds according to the injury time [3,4]. Acute wounds refer to wounds that form suddenly and heal quickly. They usually heal by primary healing in cases such as elective surgical incisions, superficial epidermal trauma, and second-degree scalds. Chronic wounds refer to skin tissue injuries caused by various reasons and the healing process takes a long time, such as ulcerative wounds, deep burns, stage III-IV pressure ulcers, diabetic foot ulcers, ulcers caused by radiotherapy and chemotherapy, etc., which are generally more than eight weeks and are easy to repeat [5,6,7]. Wounds have become one of the main causes of death worldwide, causing great inconvenience to human health and economic development [8]. Wound healing generally includes four highly integrated stages which are hemostasis, inflammation, proliferation, and remodeling [3,9,10]. These four stages start in a definite order and last for a period of time, and there may be a partial overlap in time and space between these stages. Creating a clean, moist environment for the wound can accelerate the healing of the wound without inflammation. The ideal wound dressing should have the following characteristics: maintain high humidity; remove excess wound exudate; allow heat insulation; allow gas exchange; fit the wound surface; antibacterial; no fiber shedding/non-toxic, non-adhesive, comfortable, and compliant [11,12,13]. 

Wound dressings are essential for wound healing as they provide a physical barrier between the wound and the external environment to prevent further injury or infection [3]. Traditional dressings such as gauze may adhere to the newly grown granulation tissue and cause pain when removed. In addition, it has no antibacterial, antioxidant, or other active functions [14]. Therefore, there is a need for biodegradable wound dressings based on bioactive materials that can induce wound healing and promote the deposition of extracellular matrix (ECM). Hydrogels, as hydrophilic gels with 3D network structures, generally have good biodegradability, biocompatibility, adhesion, air permeability, and maintain a moist environment for cell migration, which can effectively promote cell proliferation and facilitate wound healing [15]. These characteristics described above make hydrogels an ideal candidate product for wound dressings [16]. The multifunctional hydrogel wound dressings (such as antioxidative, antimicrobial, and injectable) currently developed can not only provide physical protection and maintain moisture in the microenvironment, but also improve the healing process by affecting the stage of wound repair [17]. For example, antioxidant hydrogels can remove excessive reactive oxygen species in chronic wounds to reduce oxidative stress, thus improving the wound microenvironment, promoting collagen synthesis and re-epithelialization, and reducing the pH value of the wound to accelerate healing and reduce infection [18]. Hydrogels fall into two categories: chemical (permanent) hydrogels that are cross-linked by covalent bonds and physical (reversible) hydrogels that are cross-linked by secondary bonds. Hydrogels can be prepared from natural biomaterials, such as alginate, chitosan, hyaluronic acid, etc.; or synthetic materials, such as polyvinyl alcohol, polyacrylamide, polyethylene glycol, etc. [19,20]. Therefore, this article reviews the physiological process of wound healing, and it focuses on the preparation methods of hydrogel dressings and the characteristics of hydrogel wound dressings made of natural biomaterials and synthetic materials. Finally, the development prospects and challenges of hydrogels are discussed.

## 2. The Physiological Process of Wound Healing

The skin is the main external defense system, which protects the body from microbial invasion and the influence of the external environment. Therefore, skin damage can pose a serious threat to human health [12]. The skin is composed of three tissue layers: the epidermis, dermis, and hypodermis [21]. The most important function of the epidermis is to form the external barrier of the body, as well as having absorption and immune functions. The dermis contains fibroblasts, mast cells, lymphocytes, and so on. Fibroblasts can produce collagen fibers, elastic fibers, reticular fibers, and matrices. At the same time, it is the main tissue repair cell after the deep damage of skin tissue. The hypodermis has loose tissue and rich blood vessels, which have the functions of connecting, buffering mechanical pressure, storing energy, and maintaining heat preservation. Knowing the composition of normal skin is helpful to our understanding of wound healing. Globally, chronic wounds impose a significant burden on patients and healthcare systems. Chronic wounds are susceptible to bacterial invasion, which can form biofilms at the wound site and inhibit the proliferation of endothelial and epidermal cells. Serious cases can be life-threatening [22,23].

**Figure 1:**
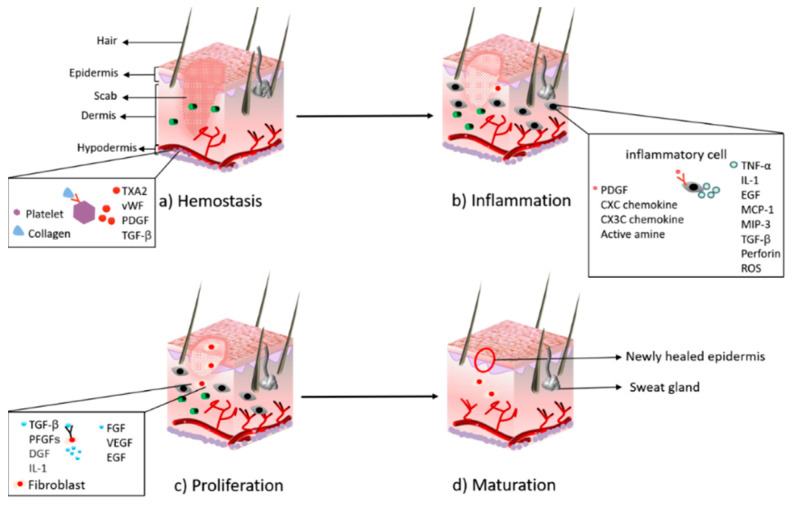
The major stages during the process of wound healing: (**a**) hemostasis; (**b**) inflammation; (**c**) proliferation; (**d**) maturation.

The wound includes the surface, the base, the cavity, and the wound margin, which is collectively called the wound bed [24]. After the body is injured, various coagulation factors play a role to achieve the purpose of hemostasis; cells near the wound secrete growth factors and cytokines to attract fibroblasts, immune cells, and endothelial cells to activate the healing cascade [25]. In the stage of inflammation, the blood vessels relax, thereby increasing the permeability of the blood vessels, and various inflammatory cells gather near the wound bed. Many inflammatory factors, such as IL-1, TNF-α, TGF-β, etc., activate lymphocytes, monocytes, and macrophages to clear pathogens and debris, and prepare for the formation of granulation tissue. A large number of growth factors and cytokines are released to promote cell proliferation and migration [15]. During the proliferation phase, many growth factors, such as FGF, VEGF, EGF, play an important role in promoting cell proliferation. The endothelial cells grow rapidly and induce the formation of blood vessels in the granulation tissue, the damaged tissue is gradually replaced by epithelial cells and fibroblasts, and the wound surface is gradually filled with granulation tissue. Maturation is the final stage in which connective tissue is formed and the new epithelium is strengthened [26,27]. Figure 1 shows the physiological process of wound healing.

## 3. Preparation Method of Hydrogels with a Promising Treatment for Wound Healing

**Figure 2:**
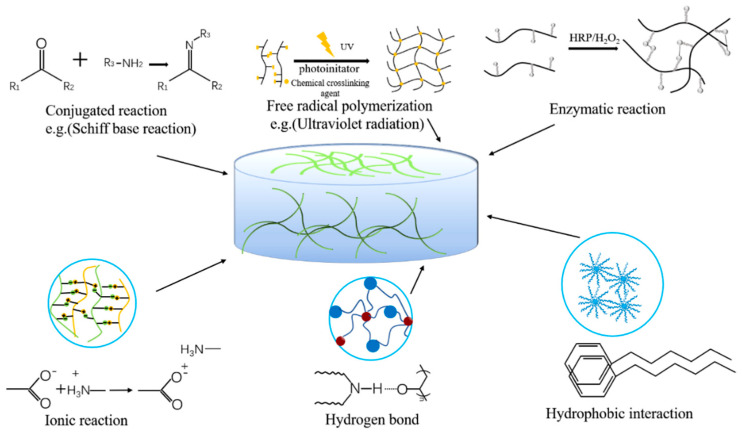
Cross-linking methods of hydrogels.

Different hydrogel wound dressings are selected according to different wound conditions. An ideal hydrogel wound dressing should have the following characteristics: (i) it should have biocompatibility and blood compatibility, and hydrogels should be able to stop bleeding immediately and stimulate hemostasis-related factors to act to promote wound healing [28]; (ii) hydrogels should have sufficient adhesion and excellent mechanical properties, even under humid and dynamic conditions they can adhere to and completely seal wounds to prevent bacterial infections [29]; (iii) good moisture retention, providing moisture to the wound site, maintaining a moist environment for cell migration, and promoting cell proliferation [30]; (iv) it can be completely degraded after a period of time, and no by-products are produced [31]. At present, the preparation of hydrogels is mainly divided into physical cross-linking and chemical cross-linking. The effect of cross-linking determines the physical and chemical properties and functions of the hydrogel dressing. Figure 2 shows some preparation methods. The wound microenvironment is affected by many factors, and hydrogel characteristics play a major role in maintaining a favorable microenvironment. According to the required conditions, different preparation methods are selected [32].

### 3.1. Physical Cross-Linking

Most hydrogel dressings with toughness and a high self-healing ability are generally formed by non-covalent bond polymerization. The 3D network structure of the hydrogel dressing formed by physical cross-linking is mainly formed by the interaction between molecules. 

#### 3.1.1. Ionic Interaction

Based on the dynamic interaction between oppositely charged groups or metal-ligand interaction is an effective way to carry out ionic interactions [33]. The hydrogels formed by ionic interaction have good ionic conductivity, fatigue resistance, environmental response, and self-healing ability. However, the poor mechanical properties and complex preparation process of hydrogels formed by ionic interactions are still the main problems that prevent their further application [31]. At present, more and more researchers are focusing on designing new hydrogels to solve these problems. For example, negatively charged monomer acrylic acid (AAC) and positively charged 2-hydroxypropyl trimethylammonium chloride chitosan (HACC) through ion interaction to form a high-density dynamic ionic bond of the compact structure of PAAC/HACC hydrogel. The structure endows the hydrogels with good mechanical properties, ionic conductivity, and good self-healing properties, the ionic conductivity is adequate to transfer bioelectrical signals and electrical stimulation on the cell proliferation and differentiation in the human body [34]. Liu et al. prepared CNF/G/Ag0.5 interpenetrating polymer network hydrogels (IPN) through dynamic ionic bond cross-linking. The hydrogels adhered to the surface of the wound and led to platelet aggregation. Gelatin can promote the erythropoiesis, and increase the number of platelets and white blood cells to blockade bleeding and the moderate cross-linked hydrogels could increase water absorption efficiency and decrease the water vapor diffusion, and as a consequence, decreased the WVTR to keep an appropriate balance of fluids on the wound bed [35].

#### 3.1.2. Hydrogen Bond

The use of hydrogen bonds is often indispensable, and the self-repair and self-recovery capabilities of hydrogels can be greatly improved through hydrogen bonds [36]. However, because hydrogen bonds are often unstable in aqueous environments, the resulting hydrogels often have low utilization rates. Currently, researchers have improved the effect of hydrogen bonding by preparing DN hydrogels or IPN hydrogels [37]. Bi et al. constructed physically cross-linked chitosan-polyvinyl alcohol DN hydrogels based on multiple hydrogen bond interactions. Since the hydrogen bond interaction is a dynamic interaction, the hydrogels can be spontaneously rebuilt after being destroyed. In addition, the hydrogels prepared by physical cross-linking have good cell compatibility and biodegradability [38]. Zhao et al. used hydrogen bonds to promote the self-assembly of SA in the PAM porous matrix. PAM-SA semi-interpenetrating polymer network hydrogels not only have good mechanical properties but also have good self-healing efficiency at room temperature based on hydrogen bond interaction. This property can prolong the hydrogels’ lifespan during their applications, especially in a severe environment [39].

#### 3.1.3. Crystallization

The freeze–thaw method is one of the commonly used methods of physical cross-linking. In the freezing part of the cycle, the formed ice crystals arrange the polymer chains around themselves. Then, during the thawing process of the cycle, the ice crystals melt to form a microporous structure [40]. The time, temperature, number of cycles, and the content of polymer components can be controlled during the freeze-thaw process for different pore sizes, mechanical strengths, morphology, or other characteristics [41,42]. The soft, flexible, and variable pore hydrogels prepared by the freeze-thaw method can simulate ECM, and the stem cells mounted on it can sense and respond to the dynamic changes of ECM stiffness, and respond and move in a directional manner, which is crucial for recruiting cells for wound healing [43]. By changing freeze-thaw conditions, PVA/HA hydrogels with a wide range of stiffness spectrums can be useful dressings for basic research related to stem cell differentiation, reprogramming, cell migration, and tissue regeneration [44]. The study of Bor et al. showed that increasing the number of F/T cycles will cause the PVA hydrogels to become hard, thus facilitating the release of drugs, which in turn accelerates wound healing [45].

#### 3.1.4. Hydrophobic Interaction

Hydrophobic interaction is a strong and stable physical interaction, which is a method of cross-linking hydrogels in water-soluble polymers with hydrophobic end groups, hydrophobic side chains, or hydrophobic monomers [46]. The mechanical properties of hydrogels can be improved by incorporating hydrophobic units into hydrogels by chemical or physical methods [47]. Su et al. introduced hydrophobic alkyl groups into PAAm and PAAc hydrogels to enhance the adhesion of hydrogels so that the hydrogels will not easily fall off from the wound due to sweating and rubbing [48]. The use of hydrophobically modified gelatin (HMG) as a hydrogel material has great potential as a carrier for charged hydrophilic therapeutic drugs (bFGF) and hydrophobic drugs (fluorescein sodium). As hydrophobic adsorption is reversible, drugs should be gradually released from HMG hydrogels in vivo to satisfy the adsorption equilibrium because released drugs diffuse out into the body fluid, thereby promoting angiogenesis [49]. The mechanical properties of hydrogels significantly affect cell spreading, proliferation, and differentiation. However, the method of adding hydrophobic units to increase the mechanical strength usually sacrifices the ductility of the hydrogels. Therefore, the development of mechanically enhanced hydrogels with coordinated elongation and toughness is still a topic worthy of attention. 

#### 3.1.5. Protein Interaction

In the preparation of hydrogels with natural polymers as the main raw materials, more and more proteins are used, such as gelatin, collagen, silk fibroin, matrix glue, and so on [12,50]. Through non-covalent bond interactions between proteins or polypeptides, conditions such as temperature and phase transition are changed to form protein or polypeptide hydrogels. Spider silk utilizes the sequence differences between eADF3-CTD and eADF4-CTD to self-assemble into β-sheet-rich silk. The precise molecular abundance and composition allow fine-tuning of the solution-gel transition process [51]. With the continuous development of protein engineering, the application range of protein-based hydrogels has become wider and wider, especially the application of recombinant proteins, such as recombinant human collagen (RHC). Deng et al. used RHC conjugated with chitosan to form thermally responsive hydrogels. Experimental results showed that the hydrogels combined with RHC exhibited greater cell infiltration capacity, and could induce more blood vessel formation and accelerate wound healing [52]. Interestingly, both the cell proliferation rate and cell morphologies were found to depend on the hydrogel composition. Increasing the RHC fraction of the hydrogels gradually enhanced the cell spreading and proliferation rate [53]. 

### 3.2. Chemical Cross-Linking

At present, most of the hydrogel materials are prepared by chemical cross-linking. Chemically cross-linked hydrogels often have good mechanical properties and stronger stability [54]. They are dominated by the conjugation reaction, free radical polymerization reaction, and the enzymatic reaction.

#### 3.2.1. Conjugation Reaction

The hydrogels cross-linked by the conjugation reaction have become a hot spot. The conjugation reaction can be carried out under relatively mild conditions, including the Michael addition reaction, the Schiff’s base reaction, and the Diels–Alder addition reaction [55]. The Schiff’s base reaction (the condensation of amine and active carbonyl group) is a simple green method in the conjugate reaction [56]. Many polysaccharide molecules contain adjacent hydroxyl groups, such as alginate, starch, hyaluronic acid, and cellulose, which can be oxidized by periodate to form hydrogels through Schiff’s base reactions [29,57,58]. Using oxidized hydroxyethyl starch (O-HES) and modified carboxymethyl chitosan (M-CMCS) as raw materials, an injectable in-situ hydrogel with excellent self-recovery, biocompatibility, biodegradability, and transparency was prepared by the Schiff’s base reaction, which can be injected into irregular-shaped skin defects and formed in situ to shape the contour of different dimensions. The excellent compliance made hydrogels easy to adapt to the wound under different conditions of skin movement, and full-thickness skin defects treated with M-CMCS/O-HES hydrogels demonstrated a higher wound closure percentage, more granulation tissue formation, faster epithelialization, and decreased collagen deposition. It is a promising therapeutic strategy for wound healing [59]. In addition, a novel bioadhesive hydrogel with intrinsic antibacterial properties was prepared by mixing modified hyaluronic acid (HA) and ε -polylysine (EPL) using the Schiff’s base reaction, which can effectively kill bacteria on the surface of wounds, promote angiogenesis, and accelerate wound healing [60]. The QCS/PF hydrogel prepared by the Schiff’s base reaction has antibacterial, antioxidant, hemostatic, adhesion, and mechanical adjustable properties. The gel promotes blood coagulation and the synthesis of ECM components by simultaneously down-regulating TNF-α and up-regulating VEGF, promotes cell signal transduction through electrical stimulation, clears ROS through curcumin, and prevents infection through its antibacterial properties. So as to promote the progress of wound hemostasis, inflammation, and remodeling stages [13].

#### 3.2.2. Free Radical Polymerization

Free radicals can be produced by heating, ultraviolet radiation, high energy radiation, electrolysis, and plasma initiation [61]. The radical polymerization reaction of thermally initiated polymerization and light-initiated polymerization utilizes unsaturated functional groups or photosensitive functional groups to undergo free radical polymerization or cross-linking under the action of heat or light to form covalent bonds [60,62]. Most of the hydrogels prepared by thermally induced cross-linking reactions can be used for a deep wound healing treatment, and the structure is stable and highly controllable [60]. In the photo-initiated polymerization reaction, the precursor containing the photosensitive functional group can be directly polymerized under UV radiation, and the precursor containing the double bond functional group can be polymerized under UV radiation by adding a photo initiator [63]. At present, gelatin, starch, chitosan, sodium alginate, heparin, hyaluronic acid, and other natural polymers are prepared into hydrogel dressings through free radical polymerization, which is widely used in wound dressings. A versatile poly(acrylamide) cellulose nanocrystal/tannic acid–silver nanocomposite (NC) hydrogel integrated with excellent stretchability, repeatable self-adhesion, high strain sensitivity, and antibacterial property, was synthesized via radical polymerization at an ambient temperature. These were merited for the hydrogels to be assembled into a flexible epidermal sensor for long-term human–machine interfacial contact without concerns about the use of external adhesive tapes and bacterial breeding [64]. In situ, PAM/SA/Ag hydrogels were prepared by using silver ions in the presence of ammonium persulfate to catalyze free radical polymerization. Histocompatibility experiment results showed that the hydrogels showed higher expression of CD31 and VEGF, which are related to angiogenesis in wound healing [65].

#### 3.2.3. Enzymatic Reaction

The enzymatic reaction is the cross-linking of natural polymers catalyzed by enzymes such as transglutaminase, tyrosinase, urease, and horseradish peroxidase (HRP) [60,66]. Enzymatic reactions occur under mild conditions, which can prevent the loss of biological activity and rapid gelation, and no harmful substances are produced. At present, the use of enzymatic rapid gelation to prepare antibacterial hydrogels is promising [67,68]. Using HRP and H_2_O_2_ to explore the immobilization of low molecular weight hyaluronic acid (LMWHA) derivatives within gelatin-based hydrogels to stimulate the migration of ECs. The result shows that the enzymatic immobilization of LMWHA-Ph within gelatin-based hydrogels represents a promising approach to promote ECs’ motility and further exploitation for vascular tissue engineering applications [69]. Scientists are paying more and more attention to the 3D cell culture of hydrogels, creating a hydrogel network with reversible stiffening/softening capability is important, enzymatic reactions can afford substrate specificity and mild/predictable reaction kinetics [70]. Using transglutaminase to mediate the covalent attachment of HA and PEG macromers, the hyaluronic acid hydrogels formed in situ can specifically regulate the cell phenotype by adjusting their own mechanical and biochemical properties [71].

## 4. Biomaterials for Preparing Hydrogels

**Figure 3:**
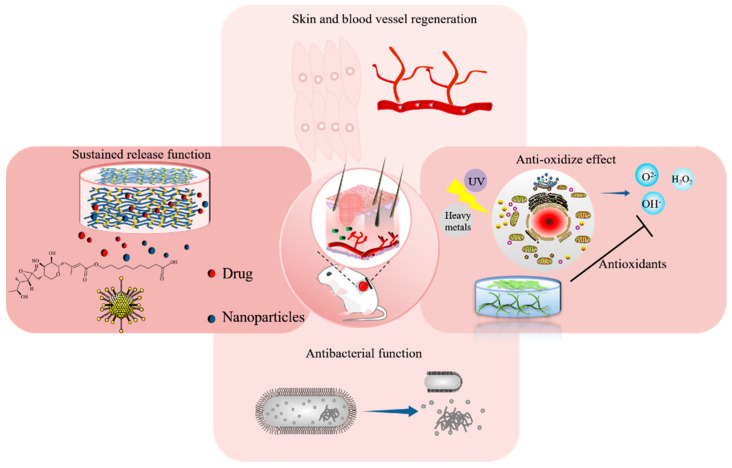
Schematic illustration of hydrogels’ function for wound repairing.

Biomaterials used in tissue engineering or regenerative medicine are generally divided into two categories: natural biomaterials and synthetic materials [72]. Regardless of the source of materials, hydrogel dressings should have low toxicity, good biocompatibility, and facilitate the growth of cells near the wound. In addition, synthetic hydrogel dressings should also have good mechanical properties, biodegradability, moisture retention, antibacterial, antioxidant, non-adhesion, and good air permeability, etc., [73] as shown in Figure 3. Recently, the hydrogel dressings synthesized by natural biomaterials have become the focus of research [74]. Natural biomaterials such as chitosan, collagen, starch, cellulose, sodium alginate, and hyaluronic acid are widely used in the synthesis of hydrogel wound dressings. However, there are some problems such as low mechanical properties, high acquisition cost, small output, and difficult modification. Synthetic materials have specific functions, good mechanical properties, large output, low cost, and rich variety. However, synthetic polymers often lack biological and biodegradable activity and may produce toxic by-products during the reaction, which lead to tissue necrosis. Therefore, researchers are committed to continuously optimizing the performance of synthetic materials and developing hydrogels with different functions.

### 4.1. Natural Biomaterials

#### 4.1.1. Sodium Alginate

Sodium alginate (SA) is a linear anionic polysaccharide polymer [75]. Because SA contains a large amount of -COO^−^, SA has obvious pH sensitivity and can quickly form a gel under extremely mild conditions, which can avoid the inactivation of active substances such as sensitive drugs, proteins, cells, and enzymes. Many wound care products take advantage of the structural similarity between alginate and ECM [20,75,76]. Chen et al. obtained the OSA-DA-PAM hydrogels through the cross-linking of hydrogen bond and dynamic Schiff’s base reaction between dopamine grafting oxidized SA (OSA-DA) and polyacrylamide (PAM) chains. Excellent cell affinity and tissue adhesiveness are necessary for the hydrogels to integrate with the wound tissue in applications. Due to lots of catechol groups on the OSA chains, the hydrogels had unique cell affinity which promoted the propagation of fibroblasts, and tissue adhesiveness, benefiting its further application in wound dressing [29]. In addition, anti-inflammatory and angiogenesis play an important role in wound healing. The emergence of antibiotic-resistant pathogens has made the problem of wound treatment more difficult [77]. In vivo evaluation of the prepared PVA-SA membrane in a mouse burn wound model induced by methicillin-resistant staphylococcus aureus (MRSA) confirmed that the coated PVA-SA membrane had the potential to control drug-resistant bacterial infections and promoted wound healing [78]. Currently, green synthesis inspired by proteins has attracted much attention. SA/SE-AgNPs semi-interpenetrating network hydrogels not only have good antibacterial activity but also overcome the shortcoming of SA lacking cell adhesion ability [79]. At an early stage of wound healing, IL-6 and IL-10 are responsible for the recruitment of fibroblast as well as the removal of extracellular matrix debris, production of pro-inflammatory molecules should subside after the inflammatory phase. The synergistic effect of each component of HG-Ag-EGCG hydrogels dressing not only accelerated the synthesis of various cytokines at the wound site, but also inhibited the survival of bacteria and excessive ROS and RNS molecules, so that the wound can transition smoothly to the proliferation and remodeling phase, thereby enhancing the repair process [80]. SA hydrogels are used as the main ingredient, embedded AgNPs, anti-inflammatory drugs, etc. to promote wound angiogenesis and inhibit bacterial production. However, the ensuing problems of the controlled release of drugs and toxicity are still major obstacles, and the preparation of hydrogel dressings still needs to be further optimized.

#### 4.1.2. Collagen

Collagen (COL), as the main component of ECM in animals, has good biological activity, biocompatibility, and biodegradability. When used as the basic component of wound dressings, it has weak antigenicity, promotes cell growth and proliferation, promotes coagulation and avoids scar formation, etc. [81]. Col-HA hydrogels prepared by HRP covalent cross-linking not only showed good biocompatibility and biodegradability but also spontaneously promoted angiogenesis and played a positive role in the formation of epithelium and collagen fiber [82]. The hydrogels with self-healing ability as a wound dressing can extend the use time of the material, especially in critical situations to provide better wound protection. Therefore, the development of high-grade collagen hydrogels with good self-healing ability, injection ability, antibacterial, and hemostatic functions as a wound dressing have great application potential [83]. Natural collagen has poor mechanical properties and weak resistance to biodegradation. Moreover, pure collagen is easy to deteriorate due to bacterial erosion in a humid environment [84]. Therefore, researchers are currently investigating a variety of cross-linking methods to improve the ability of collagen-based hydrogels. The hemostasis effect of EDC/NHS cross-linked collagen sponge was better than slow hemostasis [85]. Based on HLC and CS, HCD hydrogels were synthesized by cross-linking dialdehyde starch. Various in vitro and in vivo evaluations show that HCD hydrogels have good biocompatibility and biodegradability, indicating that HCD may be a useful cosmetic dermal filler [80]. The antibacterial hydrogel dressing was prepared from marine fish scales made of aminated collagen with low immunogenicity and high biocompatibility, OSA, and antimicrobial peptides. The developed hydrogel dressing not only exhibited a similar strain to the human skin but also can significantly inhibit the growth of *S. aureus* and *E. coli*, promote cell proliferation and migration, and accelerate angiogenesis. Moreover, the findings of the work suggested that the fish scale collagen can be represented as a promising candidate for biomedical materials, which are eco-friendly, low-cost, and sustainable [86].

#### 4.1.3. Starch

Starch is widely used in the preparation of biodegradable hydrogels [87]. It has the advantages of low cost, wide sources, renewable, biocompatibility, and non-toxicity, etc. [88,89]. Due to the shortcomings such as lack of hydrophilicity and low mechanical strength, starch is generally not used alone in the preparation of hydrogels [90]. Oxidized starch is one of the important modified starches. Oxidized starch has the characteristics of low viscosity, high stability, transparency, film formation, and viscosity, and is widely used in the pharmaceutical industry [91]. Using oxidized starch as a cross-linking agent, the gelation time and pore size of porous collagen-based hydrogels can be controlled by adjusting the degree of oxidation of starch, which is conducive to the aggregation and growth of adipose stem cells (ASCs), thereby inducing the secretion of VEGF and FGF-2 and promoting angiogenesis [92]. The oxidized starch/ZnO nanocomposite hydrogels prepared by Hassan et al. show good swelling ability and antibacterial properties and have the potential to be used in biomedicine [93]. However, the current research on hydrogel dressings with starch is not deep enough, and the low mechanical strength of starch is the main reason that restricts its further development. 

#### 4.1.4. Cellulose

Natural cellulose is the most widely distributed and abundant polysaccharide in nature, and its source is very rich. It can be produced by plants, fungi, algae, and bacteria [94,95]. Carboxymethyl cellulose (CMC) has received special attention due to its high abundance, good transparency, and low cost. CMC is highly hydrophilic, which makes the CMC hydrogels highly absorbable to wound exudate and also provides a moist environment around the wound to prevent tissue loss of moisture, which is important for burns and diabetes wounds [96]. Relatively poor cell adhesion, antibacterial activity, and water stability of CMC hydrogels have limited the practical application as wound dressings [97]. In order to make up for this defect, the blending of CMC with other polymers is the key to solving the problem. Chen et al. used Schiff’s base reactions to produce injectable hydrogel composites based on cellulose and pH sensitivity. This resultant injectable hydrogel composite system may meet clinical needs by offering localized delivery of hydrophobic compounds in a precisely controlled and environment-triggered release [98]. Compared with plant cellulose, bacterial cellulose (BC) has better properties, such as good biocompatibility, high porosity, good air permeability, moisture absorption, water retention, excellent mechanical properties, and flexibility [99,100,101]. However, BC does not have antibacterial activity. Researchers are very interested in using antibacterial agents to functionally modify them to develop new BC-based functional biomaterials for wound healing applications [102]. The thymol-rich bacterial cellulose hydrogels can be used as an effective material for third-degree burn wound repair. It not only showed excellent antibacterial activity but also had remarkable moisturizing properties that provided an apt moist environment for a smooth transition between inflammatory-proliferation-remodeling phases [103]. Compared with pure BC hydrogels, the amine-grafted BC/SPG DN hydrogels can inhibit the growth of *S. aureus* and *E. coli*, stimulate the proliferation of human fibroblasts, create favorable conditions for different stages of wound healing and promote rapid wound healing [104]. The ability of BC can be improved by compounding with other polymers to form composite hydrogels or modifying BC. Therefore, BC has great potential in the application of wound dressings in the future.

#### 4.1.5. Chitosan

Chitosan (CS) is the only natural cationic polymer with biocompatibility, biodegradability, non-toxic, antibacterial, antifungal, and anti-tumor properties. It is widely used as a biomedical material. CS is also beneficial to wound healing because it promotes hemostasis and accelerates tissue formation. CS-based hydrogels play an important role in wound healing [3,105,106]. Ding, et al. developed self-healing hydrogels based on COL and CS through dynamic imine bonding. The addition of COL makes the COL-CS hydrogels injectable have rapid self-healing ability and good moldability. In addition, the pH sensitivity of the COL-CS hydrogels are helpful for the design of smart materials [107]. The HLC/HA/CCS hydrogels prepared by using transglutaminase as a cross-linking agent greatly improves the ability of the hydrogels to act as a bacterial barrier [16]. Long et al. demonstrated the feasibility of 3D printing CS-PEC hydrogels. The 3D printed dressing showed self-adhesion to the skin. This strength of adhesion can be easily peeled off without causing tissue damage or pain. Thus, the replacement process can minimize tissue damage or scarring [108]. In addition, stem cell therapies are becoming more popular. The interactions between stem cells and materials are fundamental to stem cell behaviors including cell migration, differentiation, and self-renewal. As an important factor of the cell biophysical environment, the mechanical properties of materials can largely affect stem cell behaviors at both spatial and time scales. However, poor mechanical strength limits the application of CS hydrogels in wound healing. Considering that stem cells in vivo usually experience dynamically changed mechanical microenvironments, it will be of particular interest to further investigate stem cell mechanical memory in native-like 3D cell microenvironments.

#### 4.1.6. Hyaluronic Acid

Hyaluronic acid (HA) is one of the main components of the ECM, which interacts with cells through receptors on the plasma membrane (such as CD44) to promote the formation of capillaries [109]. During the inflammatory period of wound healing, HA accumulates in the wound area and regulates the synthesis of pro-inflammatory cytokines by regulating inflammatory cells to swallow invading microorganisms and induce fibroblasts and keratinocytes to migrate and proliferate during the proliferation and remodeling stages. Therefore, HA plays an important role in tissue regeneration and angiogenesis. Researchers have used these advantages of HA in combination with other biological materials to enhance the hydrophilicity of hydrogel dressings and promote wound healing [105,110,111]. HA-based hydrogels also have some disadvantages, such as poor mechanical properties, poor structural stability, and no antibacterial effect, which may affect their specific efficacy [112]. In order to solve these shortcomings, composite hydrogels have been prepared to improve the properties of HA-based hydrogels. Jeong et al. developed a new type of HA-based composite hydrogel with CaF_2_. Compared with pure hydrogels, the cell reactions such as cell adhesion and cell proliferation of composite hydrogels were improved [113]. Liu et al. prepared HA/EPL hydrogels through HRP enzymatic cross-linking and Schiff’s base reaction. The results of the study showed that hydrogels could effectively kill the bacteria on the wound surface and accelerate wound healing, and the thickness of new skin, new microvessels, granulation tissue, and collagen density of the rats treated with the hydrogel dressing were two times higher than those of the rats treated with commercial fibrin glue [60]. Although HA-based hydrogel dressings are endowed with good mechanical properties and antibacterial activity by cross-linking polymers or metal ions, the cytotoxicity of the added materials cannot be ignored. There is a long way to go to find more efficient green synthesis methods and less toxic materials.

#### 4.1.7. Silk Proteins (and Others)

In order to achieve the best clinical effect, a suitable dressing is designed according to the type of wound. Burns or chronic wounds will produce a lot of exudates and the shape of the wound is irregular, which is easy to be infected and even life-threatening. Silk fibroin (SF) hydrogels are good candidates because they can absorb exudate from defect sites and seal different wound defects to prevent microbial contamination [12,114]. SF has biocompatibility, biodegradability, high water absorption, high oxygen absorption, low immunogenicity, and good mechanical properties, SF hydrogel can deliver small molecular weight drugs, biological agents, cells, and simulate ECM in 3D in vitro tissue models, thereby promoting wound healing. Therefore, it can be directly used as a wound dressing [115,116,117]. Currently, natural silks of spiders, silkworms, moths, bees, wasps, and lacewings are used as an inspiration for the recombinant production of silk proteins to be used for biomedical applications [118,119,120]. The recombinant partial dragline spider silk protein is a self-assembling protein derived from *Euprosthenops australis* [121,122,123]. It possesses low density, biodegradability and biocompatibility, and good mechanical properties, in addition, it has the ability to self-assemble into materials that are suitable as cell culture matrices [124,125,126]. The functionalized recombinant spider silk proteins expressed in bacteria hold great potential for the development of advanced biomaterials in the field of tissue engineering [115,127]. The wound dressing made by Chouhan et al. using recombinant spider silk protein (4RepCT) not only stimulated cell proliferation but also provided antibacterial effects, thereby improving the wound healing process [128].

### 4.2. Synthetic Materials for Hydrogels

#### 4.2.1. Acrylamide

Acrylamide (AM) and its derivatives have excellent biocompatibility, non-carcinogenicity, non-toxicity, easy processing, mechanical adjustment, accurate and controllable elastic properties, and good swelling ability [129]. The SA/PAM-Fe DN hydrogels prepared on the basis of hydrophobic interaction and ionic cross-linking had excellent self-healing properties, puncture resistance, fatigue resistance, pH sensitivity, good thermal stability, and self-healing properties [130]. Based on the good mechanical properties of PAM-based hydrogels, the application of PAM-based hydrogels in wound dressings can be promoted by adding antibacterial, antifungal, growth factors, and other macromolecules. Huang et al. prepared QCS-M-PAM hydrogels with good hemostatic, antibacterial, and adhesion properties by using QCS and matrix adhesive. QCS-M-PAM hydrogels promote vascular regeneration and reduce scarring by up-regulating the expression of growth factors and down-regulating the expression of pro-inflammatory factors [131]. A series of excellent hydrogel wound dressings can be prepared by continuously improving the synthesis method of AM-based hydrogels and taking advantage of the excellent biocompatibility of AM. Combined with natural biomaterials, nanomaterials, growth factors, etc., AM-based hydrogels can improve their antibacterial and anti-inflammatory properties, and promote the formation of blood vessels or epithelium, so as to achieve the purpose of promoting wound healing. At the same time, AM’s mechanical adjustment and precise controllability of elastic properties make it very promising in future intelligent applications.

#### 4.2.2. Polyvinyl Alcohol

Polyvinyl alcohol (PVA) is a common polymer and has good biocompatibility, solubility, non-toxicity, non-carcinogenicity, and excellent mechanical properties [132,133]. However, the pure PVA hydrogels do not have the effects of hemostasis, antibacterial, etc., and lack of elasticity and hydrophilicity. In recent years, researchers have focused on the combination of PVA with other functional components to promote wound healing. At present, a large number of materials (such as SA, CS, gelatin, oxidized cellulose, etc.) have been mixed with PVA to achieve hemostasis [134]. Hydrogels adhered to the wound surface to block the broken blood vessels and stimulate platelets to release coagulation factors, thus promoting and accelerating blood coagulation [135]. Because of the current abuse of a large number of antibiotics, many bacteria have drug resistance [136]. Kalantari et al. prepared PVA/CS/CeO_2_-NPs hydrogels using freeze-thaw technology. Not only could it absorb a large amount of wound exudate and avoid a large number of bacteria breeding, but also because of the chemical stability, anti-inflammatory, and antibacterial properties of CeO_2_-NPS, hydrogels had strong antibacterial activity against MRSA. This can help chronic and acute wounds heal quickly [137]. Wang et al. embedded Ag/TiO_2_ nanoparticles into PVA hydrogels to effectively combat antibiotic-resistant bacteria through light-induced ROS [138]. Liu et al. prepared Cu-HHA/PVA@MΦ2 hydrogels by physical cross-linking. The hydrogels can directly provide MΦ2. As the hydrogels degrade, copper ions are released to further stimulate angiogenesis, thereby accelerating the transition from inflammation to proliferation and remodeling in the wound healing phase [139].

#### 4.2.3. Polyethylene Glycol

Polyethylene glycol (PEG) is a kind of amphiphilic polymer with a wide molecular weight range [140,141]. In recent years, PEG has been widely used in wound dressings because of its non-toxicity, good biocompatibility, biodegradability, easy availability, stable activity, and low preparation cost [142,143]. However, due to the use of cross-linking agents, such as formaldehyde, glutaraldehyde, and epichlorohydrin, the prepared hydrogel dressings are cytotoxic [144]. Therefore, current researchers focus on reducing the toxicity of PEG-based hydrogels. At present, many workers use citric acid (CA) as a cross-linking agent to prepare hydrogels [145]. It was reported that CMC-PEG hydrogels were prepared by using CA as an environmental cross-linking agent, these hydrogels had great advantages in chronic wound healing [146]. At present, the difficulty in healing chronic wounds caused by type II diabetes is mainly due to its persistent infection and prolonged inflammatory period. The presence of MRSA may exacerbate wound damage. Using PEG-based hydrogels as a drug delivery carrier can greatly improve the antibacterial activity of hydrogel dressings. Masood et al. prepared CS-PEG hydrogels containing AgNPs by ion cross-linking, the results showed that hydrogels had good antibacterial activity, which could effectively prevent bacterial damage to cells, so as to promote wound healing. This work provides a novel strategy for the development of other synthetic materials as antibacterial hydrogels for the treatment of chronic wounds [147]. The functionalized PEGS-FA hydrogel dressing significantly enhanced in vivo wound healing process in a full-thickness skin defect model by upregulating the gene expression of growth factors including VEGF, EGF, and TGF-β and then promoting granulation tissue thickness and collagen deposition [3].

#### 4.2.4. Polyurethane

Polyurethane (PU) is a synthetic polymer with urethane bonds on the main chain composed of hard segments formed by diisocyanates and chain extenders and soft segments formed by polyether or polyester blocks [148,149]. PU has good biocompatibility, blood compatibility, biodegradability, low biotoxicity, chemical stability, and mechanical properties [150]. Drug-loaded hydrogels that are physically mixed or chemically covalently bonded often receive attention because of their wide range of action [151]. However, it is often difficult for hydrophilic hydrogels to load hydrophobic drugs. At present, amphoteric PU is an ideal candidate for the production of drug-loaded hydrogels [152,153]. Lucas et al. used PEG and PCL-triol to synthesize PU-based hydrogels through a one-pot method. The amphiphilic properties of PU enable hydrophobic drugs to be loaded efficiently and quickly [154]. Feng et al. synthesized PU/curcumin hydrogels by in situ copolymerization, PU-Cur hydrogels not only retain their physical and chemical properties and functions but also improves the mechanical strength of the PU hydrogels and promotes rapid wound healing [155]. However, PU hydrogels often have disadvantages such as poor hydrophilic properties leading to weak lubricating properties, and cross-linking agents or initiators required for preparation by radiation or chemical cross-linking are toxic and difficult to remove. At present, researchers use water-based PU solution (WPU) and PVA to form PU/PVA hydrogels by freezing and thawing physical methods, which improves the hydrophilicity of PU hydrogels and greatly improves the lubrication performance of PU based hydrogels [156]. The toxicity of hydrogels can be solved by embedding carbohydrates into the PU network [157]. Carbohydrates such as cyclodextrin, starch, cellulose, etc. can be used as cross-linking agents in the PU hydrogels network due to the presence of multiple hydroxyl groups that can react with isocyanate groups [158]. 

#### 4.2.5. Polyvinylpyrrolidone

Polyethylene pyrrolidone (PVP) has low toxicity, good water solubility, biocompatibility, biodegradability, heat resistance, wettability, adhesion, and film-forming properties. Therefore, it is widely used in medicine, food, cosmetics, and other fields [159]. In recent years, the impermeability of PVP to bacteria has made PVP-based hydrogel dressings more widely used. Khan et al. prepared PVP/PVA/Ag@ZnO NCs hydrogels, and the antibacterial activity experiment showed that the dressing could maximize the wound healing rate by minimizing the increased rate of infection [160]. Due to its good water solubility, PVP tends to release the loaded drugs quickly. The strong binding effect between coumaric acid and PVP can effectively promote the controlled release function of PVP-based hydrogels. This not only solves the problem of drug delivery system design caused by poor water solubility of phenolic compounds but also solves the problem of rapid dissolution of PVP-based hydrogels in a wet environment [161,162]. Marco et al. prepared a multifunctional double-layer wound dressing. PVP loaded with skin disinfectant constitutes the upper layer, and HA/PVP loaded with antibiotic ciprofloxacin constitutes the lower layer. The results showed that the combination of HA and PVP ensured a more sustained release of the antibiotic ciprofloxacin, helping to maintain a sterile wound bed during the later stages of the healing process [163]. 

#### 4.2.6. Poly(*N*-isopropylacrylamide)

Poly(*N*-isopropylacrylamide) (pNIPAM) has both hydrophilic amido groups and hydrophobic isopropyl groups on the macromolecular chain. Both the linear pNIPAM aqueous solution and the cross-linked pNIPAM hydrogels exhibit temperature-sensitive characteristics. The thermal response is a key function of pNIPAM [164]. Moreover, PNIPAM is non-toxic and has good biocompatibility/biodegradability and physiological reactivity. Nanohydrogels based on PNIPAM have been widely used in the controlled delivery of small molecule drugs and have attracted much attention in the field of hydrogel dressings [165,166,167]. Thermally sensitive hydrogels are widely used in tissue regeneration, collagen, HA, and other polymers can be used to modify PNIPAM hydrogels to improve their biocompatibility. The heat-sensitive composite hydrogels synthesized by Lin et al. gradually deliver DS and bFGF during the inflammation phase and the new tissue formation phase by controlling the temperature for wound repair [168]. The COL–GG–PNIPAMs IPN hydrogels prepared by Zhang et al. have the properties of processability, injectability, and remodeling, which significantly promoted the repair of mouse skin wounds. In addition, they are expected to be widely used in biomedical engineering, wearable electronic devices, and sensors due to their excellent thermal sensitivity and near-infrared response characteristics [169].

**Figure 4:**
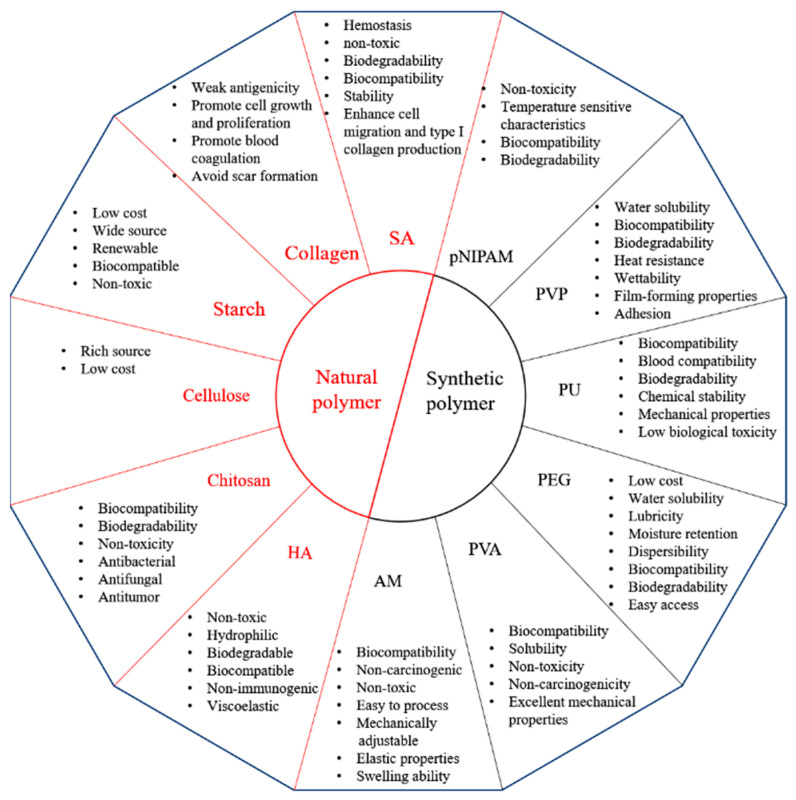
Physical and chemical properties of natural and synthetic polymers 5. The Advantages of the Clinical Use of Hydrogel Dressing.

Wound healing involves a variety of biological reactions, and the application of hydrogel dressings that do not match adjacent tissues often leads to tissue inflammation or rejection. The nature of the polymer almost determines the nature of the hydrogels made from this polymer. The properties of each polymer are shown in Figure 4. Therefore, various factors, such as mechanical properties and antibacterial properties of dressings, should be considered emphatically in practical application. However, because single polymer hydrogels often have few advantages, many researchers are working on the development of new hydrogels with enhanced physical and chemical properties, biocompatibility, controllable biodegradability, and low toxicity by combining natural and synthetic polymers or modified natural biopolymers through physical or chemical cross-linking [170,171].

The clinical outcome of wound recovery is primarily determined by the time to complete wound healing, or the proportion of wounds that fully healed (epithelialization) within a specific period of time and wound infection [172]. Hydrogel wound dressings have many benefits for wound repair. Recent progress has shown that hydrogel wound dressings can provide complex scaffolds and serve as a delivery system for cells and biochemical factors, their treatment can heal wounds faster and obtain collagen-rich and thick wound repair tissue [173]. Currently, widely used hydrogel wound dressings (eg, Vigilon, Radicare, RadiaGel, Geliperm, FibDex^®^) can quickly heal wounds [174]. Clinical experiments suggest that wounds dressed with hydrogels healed more rapidly than those dressed with a variety of usual care regimens, the average healing time of the hydrogel dressing was 13.6 ± 9.6 days, while the regular care was 15.1 ± 6.45 days, the hydrogel dressing was changed less frequently and the infection rate was lower, [175]. The research results also show that hydrogels can effectively relieve the pain, burning, and irritation typical of skin wounds. Concurrently, adverse reactions such as wound dryness, swelling, pruritus, and fever were significantly reduced [176]. In addition, the hydrogel dressing is easier to remove, the dressing replacement is simple and the cost is lower, which not only improves patient comfort but also saves resources [177]. Although hydrogels have shown superior properties in a series of in vitro and in vivo studies for wound management, they are still far from ideal wound dressings.

## 5. Conclusions and Prospects

In this review, the research progress of hydrogels as a wound dressing is reviewed and summarized. Traditional gauze and cotton dressings often cause secondary damage to the wound when they are removed. Hydrogel dressings are beneficial to the treatment of any type of wound. Researchers have prepared hydrogels with different properties through physical and chemical cross-linking methods, which greatly meet the needs of humans for the treatment of various wound types. In addition, various methods are used to continuously optimize the parameters of the hydrogels to expand the scope of application of the hydrogels. From the point of view of the materials, both natural and synthetic polymers have good biological activity and a wide range of applications. In particular, the mixed-use of various polymers highlights the main role of each component and speeds up wound healing.

In the future, the research of hydrogel dressings will develop towards lower cost and diversified functions. At present, there are various types of hydrogel dressings, and various new hydrogel materials are continuously optimized and their functions are becoming more and more perfect. However, there is still a gap with the ideal hydrogel dressing, especially in the treatment of chronic wounds. In the future, the development of hydrogel dressings can pay more attention to the preparation of materials with strong antibacterial, antioxidant, or slow-release function, or pay more attention to the structural modification of natural polymers. Many factors including materials and methods can affect the properties of hydrogels. The stability, processability, and solubility of the polymer are also important obstacles to optimizing the preparation process. Therefore, researchers can focus on searching for new technologies and materials and consider various influencing factors to improve the function of hydrogel dressings.

## Data Availability

Not applicable.
